# Planetary health at a decade: from Bellagio to practice

**DOI:** 10.1016/j.joclim.2026.100751

**Published:** 2026-10-01

**Authors:** Ann Kurth, Karin Leder

**Affiliations:** a New York Academy of Medicine, USA; b Co-lead Planetary Health Division and Head of Infectious Diseases Epidemiology Unit, School of Public Health and Preventive Medicine, Monash University, Melbourne, Victoria, 3004, Australia

**Keywords:** Planetary health, Climate change and health, Public health, Health policy, Health systems, Environmental health, Health equity, Global health, Health communication

## Abstract

**Introduction:**

In its first formally defined decade, the "planetary health" framing has had demonstrable impact but barriers to more radical action remain.

**Objectives:**

We reflect on progress during planetary health’s first decade, identify barriers limiting its wider uptake and application, and propose pragmatic communication approaches for engaging health practitioners, policymakers and the public.

**Methods:**

We have drawn on our experience as two practitioners working at the nexus of climate and health, merging our perspective with a targeted literature search.

**Results:**

In its first ten years, the field of planetary health has shaped systems thinking and shown significant impact on curricula, research, funding and publications. Possible obstacles for achieving greater transformational change include abstraction, resource intensity of transdisciplinary research, inadequate integration of Indigenous and local knowledge, politics, and academic siloes. Safeguarding of human health requires a shift to integrated, systemic thinking that resonates beyond academia, and reframing of core messages to tailor to specific health practitioner, policymaker and public audiences.

**Conclusions:**

Planetary health is a valuable integrative paradigm, but actionable cross-sectoral translation of its conceptual ambition requires pragmatic operational guidance, flexible framing, and avoidance of over-complication and political obstruction. This next decade must prioritize translation and urgent, applied policy and practice action.

## Highlights

• Planetary health has gained academic traction but complexity, terminology and governance silos impede wider engagement and action.
• Audience-specific framing could reduce abstraction and make planetary health more tangible and relevant.
• Highlighting human health co-benefits may help move ambition into solutions-focused action.

## 1 Introduction

As two recent Rockefeller Bellagio Center Residency Program recipients, we have been inspired to reflect on the progress made over the last decade in planetary health since the 2015 Rockefeller Foundation-Lancet Commission report [1]. With three planetary health convenings held at the Rockefeller location in Italy from 2014 to 2016^a^ we were honored to spend time at the place where "planetary health" was formally defined as the imperative to safeguard “the health of human civilization and the state of the natural systems on which it depends [2].” We have considered the impact this nascent field already has had in shaping transdisciplinary systems thinking, and the laudable academic traction achieved, influencing research, education, practice and policy innovation in a myriad of ways.

Noteworthy among achievements is the evolution of planetary health networks such as the Planetary Health Alliance; operationalization of curricula and healthcare frameworks; promotion of low-footprint diets; integration of Indigenous knowledge that view humans and environments as interconnected systems; expanded adoption of urban nature-based solutions; development of planetary boundaries and health policy-related metrics; emergence of dedicated funding ecosystems; and growing reference to planetary health in philanthropic and multilateral agency strategy documents [[3], [4], [5], [6], [7], [8], [9], [10], [11]]. The framing has shaped the launch of several journals, which have since become intellectual hubs for this rapidly maturing field. Publications using the explicit term “planetary health” have increased since the first handful of publications prior to the *Lancet* Report and since a 2015–2018 scoping review, although overall numbers remain relatively modest at roughly a thousand articles in English-language peer-reviewed journals [12]. It is worth noting, however, that this is a broader issue across the publication landscape, with a recent bibliometric review observing that despite climate change being the major threat to global health, many journals until recently published little on the topic [13] (Fig.).

**Fig. 1:**
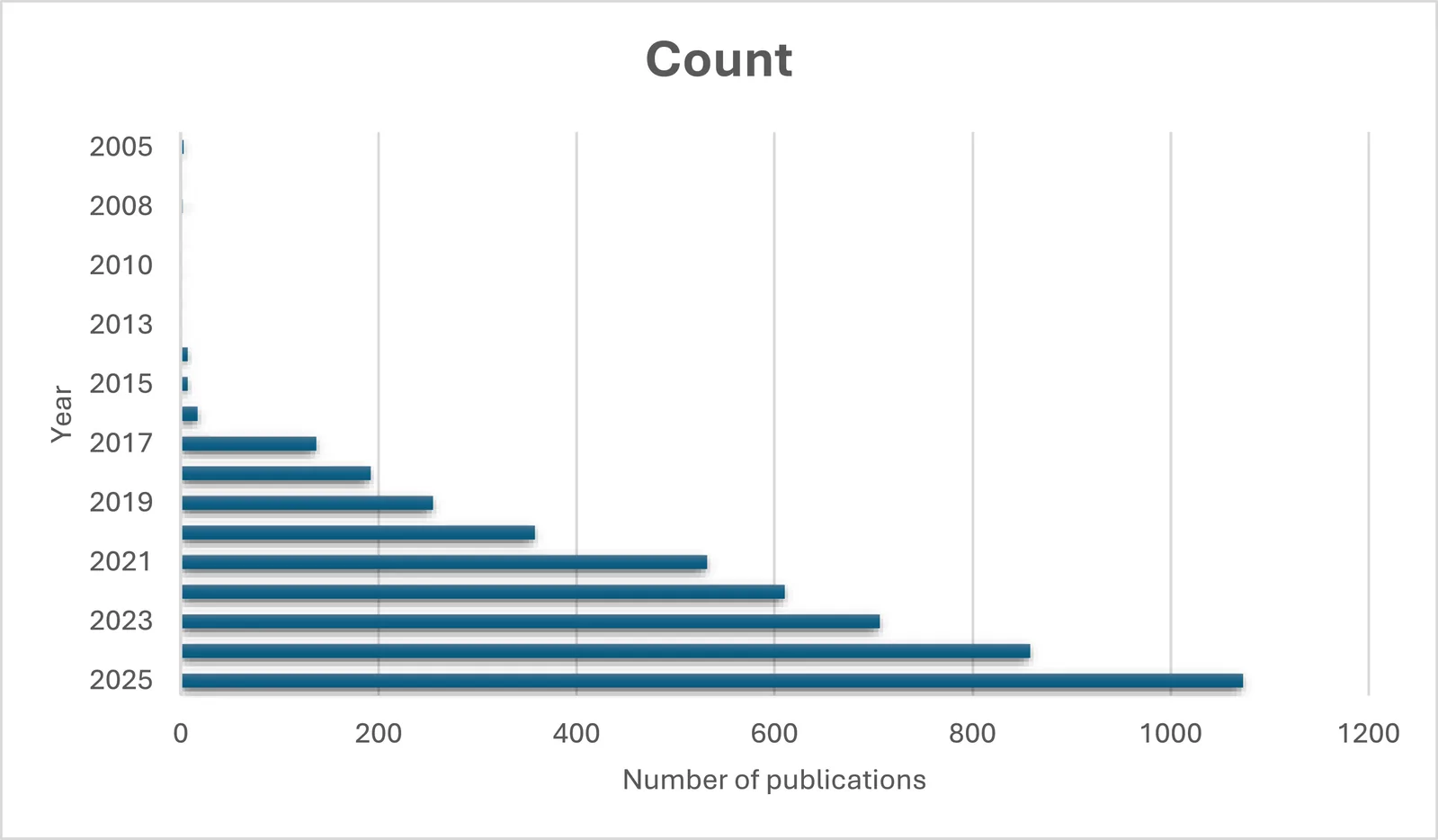
Number of publications with “planetary health”, by Year*. *This figure was created via combining references 12–13, and a literature search using the term “planetary health”.

However, we are also humbly aware that broad galvanizing of scientists and policymakers towards transformation from siloed and linear, to integrated and systemic thinking, has not been fully achieved [14]. Perhaps it is unrealistic to think that more progress could have been made over a mere 10-year period. However, given the urgency needed to address planetary health, including issues related to climate change, as well as increasing politicalization in the current moment, we have also been considering how removing barriers to more radical action might be achieved.

## 2 Pragmatic obstacles

### 2.1 Terminology

Outside specialist circles, the term "planetary health" itself is poorly understood by health practitioners, policymakers and the public. The core intention was to establish a research and policy agenda conveying the interconnectedness of earth systems, animal and human health, biodiversity loss, equity and socioeconomic factors. However, this is not intuitive or easily conveyed. A study of over 1,300 health-focused university students in Germany showed that 74% had never heard the term planetary health, and only a minority of those who had could define it clearly [15]. Planetary health is seen as extending global health, environmental health, climate change, One Health and Ecohealth paradigms by adding attention to multi-system risks, justice, Indigenous knowledge, planetary boundaries and governance [16,17]. These other frameworks do not explicitly reject these concepts but are arguably more narrowly focused. The use of the planetary health term could at times unintentionally lead to confusion and competition for attention and funding between overlapping frameworks [18].

### 2.2 Complexity and abstraction

Planetary health’s incorporation of a complex Earth-systems approach risks leading to overcomplication and abstraction. Systems thinking aims to reduce fragmentation by offering a holistic approach to problem-solving, recognizing the interconnectedness of multiple components. It emphasizes collaboration, innovation, and long-term thinking to address systemic challenges effectively. However, by incorporating this complexity, planetary health may be perceived as being overly theoretical and remote from immediate threats, thereby hindering practical action and making change more difficult to operationalize. The proliferation of glossaries and technical frameworks in planetary health literature, while clarifying, may alienate rather than empower communities and decision-makers [19]. The scope of planetary health is inherently global, which may make it seem distant and overwhelming to individuals and communities, potentially increasing the feeling that effecting change on a global scale is near-impossible. Communication science emphasizes that framing should not lead to fatalism. Instead, it should emphasize co-benefits to humans, realistic hope, and pairing with solutions and actions [20].

### 2.3 Resource intensity

Holistic planetary health research that integrates ecological, environmental, human health, animal health, socio-economic data, as well as political and governance factors, is costly, time-consuming and methodologically demanding. Multilayered data collection and interpretation is ideal, but funding for cross-sectoral work remains scarce, thus limiting opportunities for capturing comprehensive insights.

### 2.4 Integration of Indigenous and local knowledge

Indigenous knowledge is critical for legitimacy, relevance, and solution effectiveness. However, epistemic siloes and power imbalances continue to impede equitable integration into research and policy reform.

### 2.5 Short-term political focus and governance inertia

Planetary health inherently demands cross-ministerial collaboration and a global framework, whereas political decision-making often prioritizes immediacy, measurable local gains, and short time horizons, overlooking the long-term consequences of their actions on the planet. Many governments remain structured around siloed departments that are inadequately suited to tackle systemic problems.

### 2.6 Indicator gaps and academic siloes

As an inherently transdisciplinary field, planetary health brings together diverse academic, policy, and practice knowledge. Efforts to develop relevant metrics are underway, but clear operational indicators that cut across domains currently remain limited [21]. Furthermore, academic incentives that promote siloed work impede integrated, actionable outcomes.

## 3 Reframing for resonance

Despite these barriers, there remains power in the planetary health conceptualization, especially if harnessed more effectively. To resonate beyond academia, planetary health should be communicated not only as an ethical imperative but also as a practical necessity for safeguarding human health and security. If the term planetary health is inadequately understood and is seen as misaligned with political priorities (especially by partisan or time-pressed policymakers), then we should strategically reframe core messages to specific audiences [22]. No other term encompasses the needed breadth, but being less purist, disentangling the complexity of earth-systems, and showing more flexibility about labels may help drive translation and advance application of the fundamental principles (Table). We see this not as diluting, but as pragmatic, aligning with scientific advice about climate change communication [23,24]. Below we outline a strategic approach for communicating with key audiences.

**Table:** Evidence-based communication principles for planetary health.

Lead with human health now, not abstract futures Pair risk + specific solution + action Build efficacy (what works, who’s doing it, how to start) Use human-centered visuals, avoid doom imagery Pre-bunk anticipated misconceptions (e.g. “too costly”, “not local”) Keep it short, concrete, repeatable; use trusted messengers

### 3.1 Health practitioners

Foregrounding human dependence on the integrity of natural systems, while more anthropocentric than the planetary health framing that puts equal value on the health of people and the planet, may be effective. For example, *“Protecting people’s health means protecting the air, water and food systems we all depend on*” or “*Nature as our life-support: healthier people*”**.** The message is that degrading the planet’s life-support systems beyond critical thresholds makes human survival untenable, with consequent adverse impacts on human health and increasingly overwhelmed health systems. This approach involves demonstrating the causal connections between climate disruption, land-use change, biodiversity loss, and population displacement, which are already altering patterns of infectious disease, non-communicable disease, nutrition, and mental health [25]. It underscores the shared responsibility of the health sector in addressing environmental decline and improving sustainability [26].

However, health practitioners may find the term “climate (change) and health” more intuitive and may find it easier to respond to this narrower option as part of an evolution to a broader planetary health framework. This term potentially is well-suited to a clinical audience, as it is conceptually more straightforward to understand the impacts of climate – including heat, bushfires, extreme weather events (such as cyclones, hurricanes and floods) and air pollution – on individual health and on healthcare systems.

### 3.2 Policymakers

Much of what needs to be done to encourage real and rapid change sits in the realm of policymaking enacted through politicians, legislators and civil servants, that should be informed by public and patient voices [27]. Planetary health challenges should be presented as opportunities for investing in positive change, and communication should focus on the immediate co-benefits of health improvements, essential services, local resilience, economic gains, and job creation that is possible [28]. For example, *“Strengthening community resilience and health protects families, jobs and our economy”***.** This needs to be linked with concise, actionable evidence about tangible harms to health and wellbeing, and solution-focused co-benefits, which have been shown to boost support for policy [29,30].

### 3.3 The public

The public are likely to respond to clear language that transcends ideology, using messages about tangible benefits of action to their health and their communities**,** for example emphasizing clean water, healthy food, and less vector-borne illnesses [23].

The evidence base supports that this pragmatic and strategic communication shift to frame environmental change in terms of concrete health impacts rather than abstract ethical framing resonates more widely across clinical, policy, and public audiences. This may help foster progress rather than pushback. Framing environmental protection as essential for ensuring food security, safe water, clean air, climate stability, and mental wellbeing is an approach that aligns with global reviews showing that public awareness of the health relevance of climate change is limited, but informing people about both harms and benefits of action significantly boosts engagement and policy support to move from concept to action.

Sometimes metaphors are used, such as describing the planet as a patient in crisis. Communications experts caution that leaning on crisis language may make people feel overwhelmed such that they retreat to fatalism and disengage. Urgency messages can be effective but should be balanced with highlighting of real solutions and include effective and human-scaled storytelling [31]. Action steps can help support mental health responses to the planetary polycrisis [32]. Planetary health framing provides a potential "polysolution" approach, incorporating as it does systems thinking, transdisciplinarity, synergies and an emphasis on equity and scalability.

## 4 Conclusion

Planetary health, born definitionally at Bellagio, has advanced our collective capacity to align human and planetary wellbeing. In its first decade, it has shaped research, curricula, funding, and design of layered approaches. As planetary health enters its second decade, the challenge is not only to deepen systems thinking and action, but also to avoid over-complication [33]. Its very ambition risks overwhelming decision-makers, educators, researchers and communities, who instead need to feel equipped with decision tools and empowered to take action. The challenge is to retain integrated scientific ambition, while ensuring pragmatic operational guidance that emphasizes human health harms, concrete services (safer food, water, air), and co-benefits of action. We can keep the term "planetary health" as our guiding star without letting it drift into abstraction. Retaining “planetary health” as a paradigm should not obscure garnering support for practical solutions and co-benefits. Remaining flexible to terminology and framing may also help address the key issues and political obstruction.

The central message remains unchanged: the health of people depends on the health of natural systems. The next decade must ensure that this insight drives not only scholarship but also, urgent action.

## CRediT authorship contribution statement

**Ann Kurth:** Writing – review & editing, Writing – original draft, Visualization, Validation, Supervision, Resources, Project administration, Methodology, Investigation, Funding acquisition, Formal analysis, Conceptualization. **Karin Leder:** Writing – review & editing, Writing – original draft, Visualization, Validation, Supervision, Resources, Project administration, Methodology, Investigation, Funding acquisition, Formal analysis, Conceptualization.

## Declaration of competing interest

The authors declare that they have no known competing financial interests or personal relationships that could have appeared to influence the work reported in this paper.

## Footnotes

a A preceding meeting in Beijing in 2013 was referenced by David Rockefeller [34]. It has been noted that the phrase “planetary health” was used by physician ecologist Frederick Sargent II in 1972, and by Jonas Salk [35] and Friends of the Earth [36] in 1980. More foundationally, Indigenous community perspectives have long precedents of holistic knowledge related to what is now called planetary health, with expertise that must be acknowledged [[37], [38].
